# Technological Optimization of Fermented *Siniperca chuatsi* Fish Processing Focused on Formation of Garlic Clove-Structural Muscle Flakes and Flavor Profiles

**DOI:** 10.3390/foods15030460

**Published:** 2026-01-28

**Authors:** Zhangqin Lai, Mi Tang, Hai Chen, Xiaoyi Tan, Yuhao Zhang

**Affiliations:** 1 College of Food Science, Southwest University, No. 2 Tiansheng Road, Beibei District, Chongqing 400715, China; laizq0701@163.com (Z.L.); mitang0321@163.com (M.T.); chenhai2509@swu.edu.cn (H.C.); 2 Chongqing Academy of Agricultural Sciences, Chongqing 401329, China

**Keywords:** fermented *Siniperca chuatsi* fish, garlic clove-structural muscle flakes (GCMF), salt brining, volatile flavor compounds, peeling properties

## Abstract

The edible and sensory quality of fermented fish products, particularly the formation of garlic clove-structured muscle flakes (GCMF), play critical roles in consumer acceptance and consumption. Herein, aiming to obtain the optimal technical process, this study systematically explored the generation and dynamic evolution of GCMF structure of fermented mandarin fish, especially the integrity and peeling properties of GCMF, which would profoundly determine the textural properties of fish flesh. Meanwhile, flavor profiles were also concentrated during the formation of GCMF. Specifically, our results showed that the optimal fermentation conditions were 3% salt concentration and 7 days of fermentation at 7 °C. Under these conditions, the physicochemical indicators (moisture, pH, TVB-N) of the fermented fish remained within reasonable ranges and the sensory score; peeling integrity of GCMF and the texture properties reached the highest values. In addition, with the increase in fermentation time, the content of undesirable flavor compounds, especially nonanal and 1-octen-3-ol, gradually decreased. Overall, these findings provide a theoretical framework for the evaluation of GCMF structure and for understanding flavor development in fermented mandarin fish, thereby laying a foundation for improved quality control of fermented fish products.

## 1. Introduction

Mandarin fish (*Siniperca chuatsi*), a primarily carnivorous freshwater fish, is distributed in East Asian regions such as China and North Korea [1]. It is favored by consumers for its tender flesh with few bones, low fat content (1.0–1.5%), high protein (18–20%), and essential amino acids (40% of total amino acids). According to data from the China Fisheries Statistical Yearbook 2024, mandarin fish production in China reached 477,592 t in 2023. As is known, the high levels of protein, amino acids, fat, and moisture in fish create a biochemical environment that is highly susceptible to microbial invasion and endogenous enzyme activity post mortem [2,3]. These degradation processes can lead to muscle deterioration and softening, ultimately affecting the texture and quality of fish products. Currently, many effective preservation strategies have been developed to improve the quality of mandarin fish and address the growing consumer demand for high-quality products. Those technologies include refrigeration preservation, freezing preservation, salt brining, and so on. For example, Li et al. demonstrated that salting turbot muscle at appropriate salt concentrations optimizes water distribution, water-holding capacity, texture, and muscle microstructure by promoting protein swelling without excessive structural damage, thereby improving the overall quality of salted turbot products [4]. These achievements have significantly advanced the development of the mandarin fish industry and provided consumers with nutritious and delicious products.

Fermentation technology stands as one of the oldest methods for food production and preservation worldwide. It is widely applied in fish processing to extend shelf life and mitigate undesirable changes in fish muscle. Notable examples include China’s fermented mandarin fish [5], sour fish [6], Thailand’s Pla-ra [7], and Japan’s fermented fish [8]. In general, fish fermentation not only enhances product safety but also imparts unique texture and flavor characteristics. For instance, sour fish, a product of carp fermented with roasted cereals under sealed conditions, exhibited a unique flavor and higher nutritional value [9,10], and fermented mandarin fish also developed a distinctive taste, an elastic texture, and a high content of human-essential amino acids through its salting and fermentation process [11]. Interestingly, one of the most notable edible qualities of fermented fish meat is that some fermented fish products can form unique garlic clove-structured muscle flakes (GCMF), which means that the fish meat can be separated into garlic cloves after cooking. Such unique texture is deeply loved and consumed by consumers. Moreover, people often use the garlic clove-structural muscle flakes as a standard for evaluating the quality of fermented fish products, such as stinky mandarin fish [12,13]. Recently, Yang et al. found that the hydrolysis of myofibrillar and membrane structures by endogenous enzymes during fermentation are probably associated with the formation of a GCMF structure, thereby contributing to enhanced muscle tenderness and elasticity [12]. Meanwhile, it was found by Yang et al. that the fermentation of stinky mandarin fish proceeds in two stages, during which the characteristic flavor is significantly enhanced in the later phase of fermentation, and six major flavor compounds, represented predominantly by linalool and 1-Octen-3-ol, were identified as the characteristic volatile compounds [14]. Even so, most recent studies have primarily focused on the flavor control of fermented fish products [15], while the assessment methods and regulations regarding the unique characteristics of garlic clove-structural muscle flakes have been largely overlooked. Generally, traditional processing of fish meat to generate GCMF typically involves salt fermentation and pressure treatment. These processing parameters are highly associated with the intermolecular interactions between adjacent GCMF structures, thereby influencing the integrity and peeling properties of GCMF. Notably, as the most critical sensory attributes, the effects of fermentation parameters on GCMF integrity and peeling properties are largely unaddressed. Furthermore, the lack of systematic assessment methods for GCMF has significantly limited the unveiling of its generation mechanism.

Herein, this study established a quality evaluation method of garlic clove-structural muscle flakes morphology and investigated the dynamic evolution flavor profiles during the fermentation of *Siniperca chuatsi* Fish. Specifically, by developing the evaluation methods on the generation and quality of garlic clove-structural muscle flakes, we investigated the effect of salt concentration on the formation of garlic clove-structural muscle flakes and subsequent changes throughout fermentation. Meanwhile, the dynamic changes in volatile flavor compounds during natural fermentation were examined. Overall, this study establishes a theoretical basis for evaluating the salt concentration and fermentation time on the GCMF structure and flavor formation in fermented fish, identifies the optimal conditions for GCMF formation, and establishes a quality evaluation method for the GCMF. The findings elucidate the key factors that determine product quality and provide a basis for quality control in fermented fish production.

## 2. Materials and Methods

### 2.1. Materials

All processed mandarin fish were provided by Professor Luo Li from the College of Fisheries at Southwest University, Chongqing, China. A total of 48 mandarin fish, with an average mass of 500 ± 100 g, were collected and transported to the laboratory in polyethylene foam boxes with ice packs. The fish samples were rinsed under running water to remove surface mucus and blood residues, after which surface moisture were dried before further use.

Salt was purchased at Tiansheng Agricultural Market in Chongqing (Chongqing, China). Standard alkanes (C7~C40) and the internal standard 2,4,6-trimethylpyridine were purchased from Sigma-Aldrich (Shanghai, China). Chromatographic-grade n-hexane was obtained from Aladdin Industrial Corporation (Shanghai, China).

### 2.2. Preparation of the Sample

Fermentation at different salt concentrations: take 0%, 1%, 3%, and 5% NaCl concentration per fish ketone body weight, apply it evenly to the surface and inside of the fish, and place the marinated fish flat in a square stainless-steel basin (32 cm × 22 cm × 7 cm). After stacking, a 2.5 kg weight was used to squeeze the fish. The samples were fermented in a refrigerator (BD/BC-72A12BD, Hefei Royalstar Electronic Appliance Group Co., Ltd., Hefei, China) at 7 °C for 7 days. The fish were turned once daily, and the leached liquid was poured off until no additional moisture was observed. Six fish were cured at each salt concentration. After fermentation, samples were vacuum-packed and stored at −40 °C for later use. Samples were grouped into four main categories: Y0 (0% NaCl concentration), Y1 (1% NaCl concentration), Y3 (3% NaCl concentration), and Y5 (5% NaCl concentration).

Fermentation at different durations: Salted marinated fish with 3% salt by weight of fish, following the same procedure as above. The samples were fermented in a refrigerator at 7 °C. Samples were collected after 0, 3, 5, and 7 days of fermentation, with six fish per time point. Based on the sampling time, they were divided into four major groups named D0, D3, D5, and D7, representing 0, 3, 5, and 7 days of fermentation, respectively.

### 2.3. Determination of the Structural Integrity Rate of GCMF

After fermentation was completed, the head, tail, and skin of mandarin fish were removed. The backbone was carefully extracted from the dorsal side, and the axial muscles on both sides of the filet were separated along the lateral line. We placed the two filets in a steamer and cooked for 10 min, allowed them to cool until room temperature, then peeled the fillets toward the tail end. We observed the integrity of GCMF after peeling. A GCMF was judged to be intact based on visual inspection, meeting the following criteria after peeling: absence of cracks and edges that were completely intact, smooth, and without any damage.

(1) $$\mathrm{the}\;\mathrm{integrity}\;\mathrm{rate}\;\mathrm{of}\;\mathrm{GCMF}=\frac{Complete\;GCMF\;fish\;sclices\;count}{Total\;number\;of\;fish\;slices}\times 100\%$$

### 2.4. Determination of the Peeling Properties for GCMF

Use the method described in Section 2.2. to prepare the fish meat. Take the center piece of the axial muscle when the fish meat has cooled to room temperature. Measure the single-test parameters of the fish meat, such as maximum positive force, positive peak slope, breaking force, and breaking distance, using a texture analyzer (TA. XT Plus, Stable Micro Systems, Godalming, UK) after grouping the muscle fibers into sets of five. Make use of a TA 36 cylindrical probe (Stable Micro Systems, Godalming, UK) with a 36 mm diameter and a flat bottom. Set the pre-test speed to 2 mm/s, the test speed to 1 mm/s, and the post-test speed to 5 mm/s. Maintain a compression ratio of 90% and a trigger point reload of 5.0 g. Test each sample six times and calculate the average of the results.

### 2.5. Texture Profile Analysis (TPA)

Process the fish meat according to the method described in Section 2.2. Cut the fish meat into 1 cm × 1 cm × 0.2 cm pieces using Liu et al. [16] method, with the dorsal muscle. These pieces were measured using a texture analyzer (TA. XT Plus, Stable Micro Systems, Godalming, UK) in TPA mode, with parameters including hardness, chewiness, adhesiveness, and springiness. A TA 36 flat-bottomed cylindrical probe with a diameter of 36 mm was chosen. The pre-test speed was set to 5 mm/s, the test speed to 1 mm/s, and the post-test speed to 1 mm/s. The compression ratio was 50%, and the trigger point reload was 5.0 g. Each sample was tested six times, and the results were averaged.

### 2.6. Sensory Evaluation Analysis

Sensory assessments were conducted in a sensory analysis laboratory with environmental conditions compliant with ISO 8589:2009 [17], including controlled temperature (22 ± 1 °C), neutral white lighting, and an odor-free testing area. The sensory panel consisted of 10 trained members from the College of Food Science, who were recruited and trained following ISO 8586:2023 [18] guidelines.

The panel evaluated three attributes: tissue morphology, appearance, and texture. Sensory evaluation criteria were established based on Szczesniak’s methodology [19] (Table S1). An unstructured linear scale ranging from 1 to 20 conventional units (c.u.) was used, where 1 represented the lowest intensity and 20 the highest intensity of each attribute.

Fish meat was steamed for 10 min, cooled to room temperature, and then separated into GCMF for sensory assessment. Approximately 50 g of each sample was presented in odor-free white plates, coded with random three-digit numbers. Panelists rinsed their mouths with water between samples. All samples were assessed in two independent replicate sessions.

All sensory tests were approved by the Ethics Committee of the College of Food Science, Southwest University, Chongqing, China, and the permission number is HF20251201, and informed consent was obtained from all participants prior to the study.

### 2.7. pH, Total Volatile Base-N (TVB-N) and Moisture Content

The pH measurement method was performed as described by Fan et al. [20] with slight modifications. Accurately weigh 3 g of skinless fish meat and place it in 27 mL of deionized water. Homogenize it with a high-speed disperser (XHF-D, Ningbo Science Biotechnology Co., Ltd., Ningbo, China) at 8000 r/min for 1 min. The pH of the samples was then measured using a pH meter (FiveEasy Plus, Mettler-Toledo, Greifensee, Switzerland), with six parallel measurements taken per group.

TVB-N values was determined using the automatic Kjeldahl nitrogen determination method specified in the current Chinese standard [21] for the determination of volatile basic nitrogen in food.

Moisture content was determined using the direct drying method according to Gu et al. [22].

### 2.8. Determination of Volatile Compounds

Sample preparation methods were adapted from Chen et al. [23] with slight modifications. Fish meat from different fermentation days was minced into fish paste using a meat grinder (FB09B, Zhejiang SUPOR Limited by Share Ltd., Hangzhou, China). Two grams of fish paste were placed in a 20 mL vial, and 25 μL of a n-hexane solution containing 100 μg/mL 2,4,6-trimethylpyridine was added as an internal standard. Experimental conditions are as follows.

#### 2.8.1. HS-SPME Condition

After 10 min of water bath extraction at a constant temperature of 60 °C, the SPME with DVB/CAR/PDMS fiber (divinylbenzene/carboxen/polydimethylsiloxane; Supelco, Bellefonte, PA, USA) was inserted into the vial for 30 min of extraction. Following extraction, desorption was performed at 250 °C for 5 min, after which the components were separated and identified.

#### 2.8.2. GC-MS Analysis Condition

Samples were analyzed by GC-MS (ISQ 7610, Thermo Fisher Scientific, Waltham, MA, USA) following a modified procedure based on Gao et al. [6]. Gas chromatography conditions: column: TG-WAXMS, 30 m × 0.32 mm × 0.25 μm; temperature program: initial column temperature 40 °C, held for 5 min; heated at 3 °C/min to 50 °C, held for 3 min; heated at 5 °C/min to 150 °C, held for 5 min; finally heated at 20 °C/min to 230 °C, held for 5 min; inlet temperature 250 °C; carrier gas: He, 1.0 mL/min; splitless injection. MS conditions: interface temperature: 250 °C; electron energy: 70 eV; ion source temperature: 260 °C; scan range: 35–400 *m*/*z*; solvent delay time: 5 min.

#### 2.8.3. Qualitative and Quantitative Analysis of Volatile Compounds

Volatile compounds were identified based on MS spectral libraries (NIST17-1.lib and NIST17s.lib) and retention indices (RI). Quantification was performed using 2,4,6-trimethylpyridine as an internal standard. Relative quantification of volatile flavor compounds was calculated using the following formula:

(2) $$C_{n}\;=\;\frac{\rho \;\times \;V\;\times \;S_{n}\;\times \;1000}{S\;\times \;m\;\times \;1000}$$

where C_n_ represented the concentration of each volatile compound in μg/kg; *ρ* represented the mass concentration of the internal standard in μg/mL; *V* indicated the volume of internal standard added in mL; *S_n_* signified the peak area of each volatile flavor compound; *S* denoted the peak area of the internal standard; *m* represented the mass of the sample added in g; and 1000 serves as the conversion factor.

The concentration of the volatile compound was divided by its odor threshold as the OAV.

### 2.9. Statistical Analysis

Experimental data were analyzed using one-way analysis of variance (ANOVA) with SPSS 27.0 (IBM, Armonk, NY, USA), and Duncan’s multiple range test was employed for significance analysis. When *p* < 0.05 indicated significant differences experimental results were indicated. Graphs were processed with Origin 2022. Each test sample underwent three parallel replicate experiments.

## 3. Results and Discussion

### 3.1. The Effects of Salt Concentrations on the Integrity and Peeling Properties of the Garlic Clove-Structural Muscle Flakes (GCMF)

**Figure 1:**
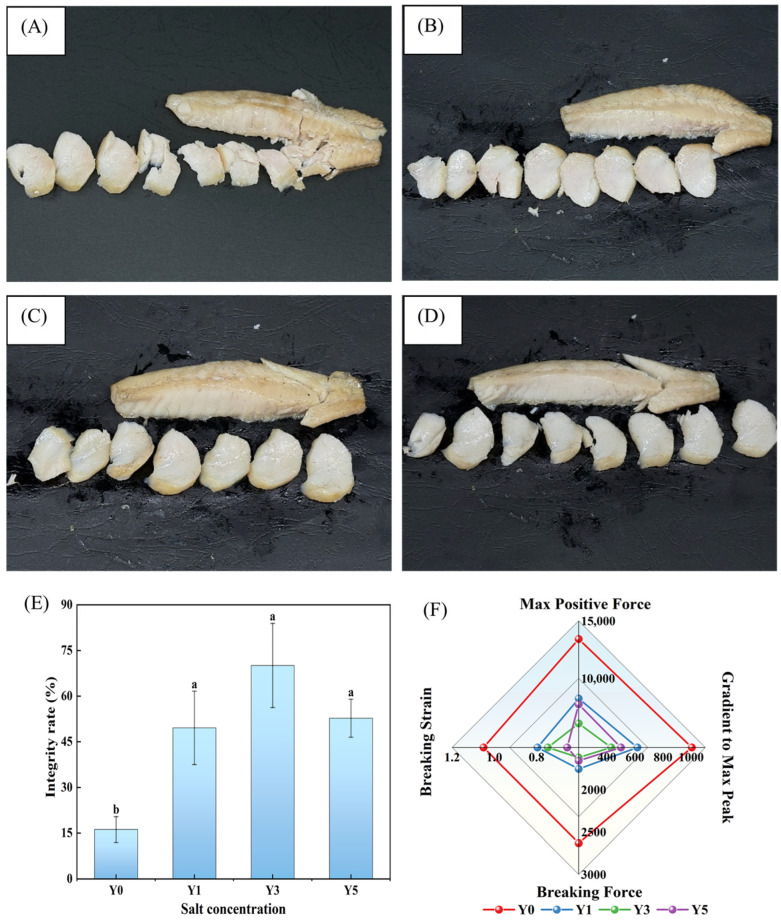
(**A**–**D**) correspond to the formation of GCMF in Y0 (without NaCl), Y1 (1% NaCl concentration), Y3 (3% NaCl concentration), and Y5 (5% NaCl concentration), respectively; (**E**) represents the effect of salt concentration on the integrity rate of GCMF. (**F**) shows the effect of fermentation at different salt concentrations on the peeling of GCMF. Different letters on the bar chart indicate significant differences (*p* < 0.05).

The morphology characteristics of epaxial musculature of mandarin fish during fermentation after heating usually shows divisive flakes, as presented in Figure 1. The fish meat flakes peeled from musculature, in the absence of salt, were incomplete and fragmented with an integrity rate of 16.20 ± 4.21% (Figure 1A,E), which is similar to the previous studies [12]. With the increase in salt concentration ranging from 1% to 5%, the formation of the garlic clove-like structure improved significantly, and the peeled fish meat flakes became more intact and the integrity rate of GCMF first increased and then decreased, reaching their highest level at 70.07 ± 13.82% when fermented at a concentration of 3% NaCl (Figure 1B–E). This could be explained by the lower moisture content of fish meat resulting from pressing and the higher salt concentration, which widens the spaces between myofibrils and makes separation easier. Persistently high salt concentration fermentation may induce abnormal swelling of myofibrils followed by dehydration and lead to the denaturation of myofibrillar proteins. This process may disrupt the cross-linked network structure between myofibrillar proteins [24,25], thereby weakening the meat structural integrity and leading to lower integrity rate for Y5 compared to Y3.

In order to further assess the peeling properties of GCMF, several parameters are applied to gain a deeper understanding of the changes during fish flake surface separation. The breaking force shows the ductility presented when fish flakes separate; the gradient to max peak shows the gradient during the pressure increase phase; the max positive force is the maximum force needed to separate the fish flakes; and the elastic deformation during separation is measured by the breaking strain [26]. Figure 1F showed that the max positive force, gradient to max peak, breaking force, and breaking strain during GCMF separation are strongly impacted by salt concentration, and the overall trend was similar to the above integrity rate properties of fish flakes. In the absence of salt, the above four parameters reached maximum values. As salt concentration increased, all four indicators reflecting the fish meat separation difficulty decreased significantly. At the 3% salting level in particular, the max positive force, gradient to max peak, and breaking force reached their minimum values, which demonstrates that a higher salt concentration reduced the cohesive force and springiness between GCMF segments, making them easier to separate. However, the 5% salt-concentrated fish flakes showed a slight increase in max positive force, gradient to max peak, and breaking force compared to the 3% salt-concentarted sample, likely due to the substantial water loss and the consequent tighter protein aggregation, which led to a stronger gel-consolidated muscle network [27].

### 3.2. The Effect of Salt Concentration on the Sensory, Textural, and Physiochemical Properties of the Garlic Clove-Structural Muscle Flakes (GCMF)

#### 3.2.1. Effect of Salt Concentration on Sensory Attribution of GCMF

Sensory Evaluation is one of the most important aspects determining the food quality [28]. As shown in Table 1, with increasing salt concentration, the organization form, appearance, and taste scores initially increased and subsequently decreased. Among the evaluated attributes, the organizational form profile refers to the degree of GCMF structural development. The appearance profile indicates surface characteristics of GCMF, and taste reflects the textural properties of hardness, springiness, gumminess, and chewiness. The organization form score increased with increasing salt concentration, indicating that the edge smoothness and integrity of GCMF were progressively improved. Accordingly, the overall morphology of GCMF became more intact and well structured, consistent with the integrity rate of GCMF. The sensory evaluation score was the lowest when no salt was added, with the appearance scoring 9.25 ± 1.71 points, the color scoring 0.38 ± 2.39 points, and the taste scoring 11.25 ± 2.49 points, respectively. In addition, the fish flakes fermentation at 3% salt concentration exhibited the highest evaluations, including visual attribute and taste. This is probably because the appropriate salt concentration can improve the appearance and taste characteristics [29]. However, the lower evaluation scores of fish flakes with 5% salt compared to the 3% group, coupled with the above texture analysis, indicate that excessive quantity of salt caused the fish meat to become overly firm and salty. Therefore, adding 3% salt is recommended to achieve the highest level of overall sensory acceptance and saltiness in fish flakes.

#### 3.2.2. Effect of Salt Concentration on Textural Properties of GCMF

**Figure 2:**
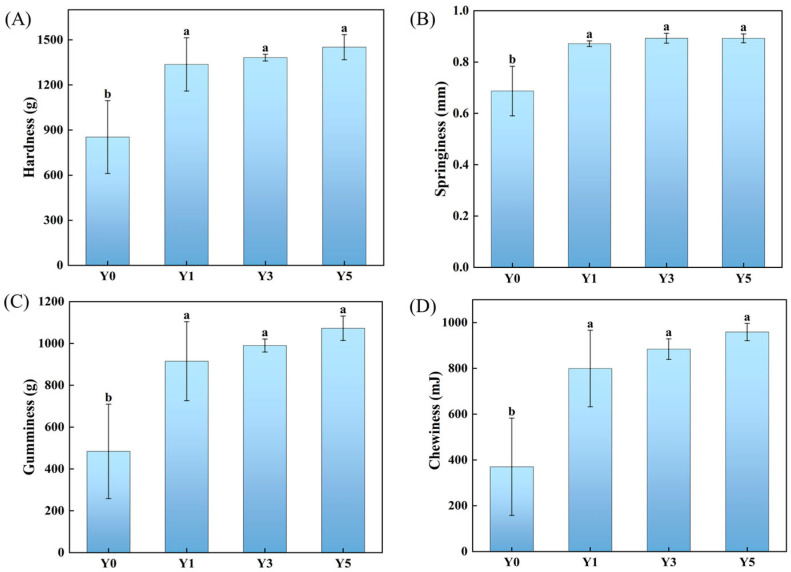
Effects of different salt concentrations on the TPA of GCMF, where (**A**–**D**) correspond to changes in fish meat hardness, springiness, gumminess, and chewiness, respectively. Different letters on the bar chart indicate significant differences (*p* < 0.05). Y0: without NaCl, Y1: 1% NaCl concentration, Y3: 3% NaCl concentration, and Y5: 5% NaCl concentration.

The textural properties of GCMF, including hardness, springiness, gumminess, and chewiness, play a vital role in assessing consumer acceptance [30]. Compared to fresh fish, the hardness, springiness, gumminess, and chewiness all increased significantly (*p* < 0.05) (Figure 2). This may be due to dehydration under external pressure. Water was transferred from the muscle fibers to the outside of the fish as an exudate. The increase in salt levels accelerated the rate of water migration and decreased the water content, causing muscle contraction [31]. Furthermore, salts could also lead to the dissolution of proteins, thereby promoting protein gel formation of GCMF [4]. Furthermore, the textural properties of the samples showed no significant changes (*p* > 0.05) with salt addition between 1% and 5%. Springiness, in particular, remained stable across this range (Figure 2B). Salt concentration exhibits an important effect on the texture of fish and promotes the occurrence of gelation and enhances the textural properties of fish.

#### 3.2.3. Effects of Salt Concentration on pH and Moisture Content of GCMF

**Figure 3:**
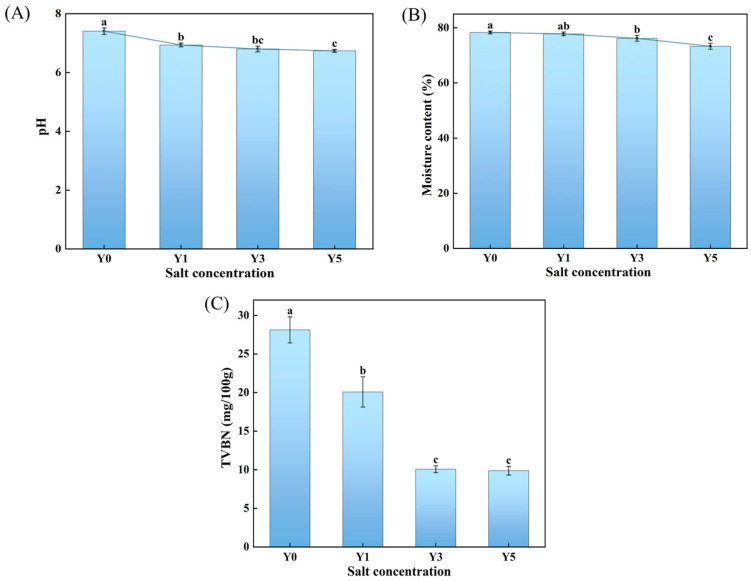
Effects of different salt concentrations on the physicochemical properties of GCMF, where (**A**–**C**) correspond to changes in fish meat pH, moisture content, and TVB-N, respectively. Different letters on the bar chart indicate significant differences (*p* < 0.05). Y0: without NaCl, Y1: 1% NaCl concentration, Y3: 3% NaCl concentration, Y5: 5% NaCl concentration.

The pH value significantly influences microbial growth, particularly that of spoilage bacteria. Figure 3A illustrated the effect of salt concentration on the final pH value of GCMF. The pH value of untreated fish meat is determined to be 7.41 ± 0.11 after 7 days of fermentation. Upon the addition of salt for fermentation, the pH value was much lower than the untreated fish meat, indicating that the salt addition can alter the final pH value of GCMF products, thereby influencing their quality and safety. Furthermore, as the salt concentration increased from 1% to 5%, the final pH exhibited a decreasing trend, suggesting that higher salt levels facilitate pH reduction. This effect may be attributed to the effective suppression of endogenous enzyme activity and microbial growth at a higher NaCl concentration under an identical fermentation duration, consequently restraining protein decomposition and the generation of amine substances. The effect of salt concentration on the moisture content of fish meat was illustrated in Figure 3B. Compared to the untreated fish meat, it is obvious that the addition of salt can result in a decrease in the moisture content of GCMF products, and a higher concentration of salt can significantly decrease the moisture content. This phenomenon occurs because salt acts as a dehydrating agent by inducing protein denaturation and disrupting the water–protein interactions. The structural changes in the proteins diminish their ability to immobilize water, consequently reducing the water retention capacity. Meanwhile, during the salting and fermentation process, the water content inside the fish muscle remains relatively high, whereas the salt concentration at the surface is elevated, forming a pronounced osmotic pressure gradient. This gradient promotes the outward diffusion of free water and part of the immobilized water from the interior of the muscle. Consequently, higher salt concentrations intensify water migration from the fish muscle, leading to a significant reduction in its moisture content.

#### 3.2.4. Effects of Salt Concentration on the TVB-N of GCMF

TVB-N refers to the alkaline nitrogenous compounds generated during the processing and storage of meat through the degradation of proteins, primarily mediated by microbial activity or endogenous enzymes. It is widely recognized as an important indicator of meat freshness [32]. As shown in Figure 3C, in the case of the untreated fish meat, the TVB-N value was measured to be 28.13 ± 1.68 mg/100 g, implying the obvious decrease in edible quality due to the growth of spoilage microorganism. The addition of salt during fermentation significantly reduced the TVB-N value to 20.09 ± 1.96 mg/100 g, 10.08 ± 0.44 mg/100 g, and 9.88 ± 0.55 mg/100 g for salt concentrations of 1%, 3%, and 5%, respectively. This reduction indicates that salt effectively inhibited the growth of spoilage microorganisms, thereby ensuring the edible quality of GCMF products. It is noteworthy that no significant differences in TVB-N values were observed between 3% and 5% salt concentrations. This suggests that a 3% salt concentration is sufficient to exert a strong antibacterial effect, a finding consistent with reports for most fermented meat products [33,34].

### 3.3. The Evolution of the Integrity and Peeling Properties of the Garlic Clove-Structural Muscle Flakes (GCMF) During Fermentation

**Figure 4:**
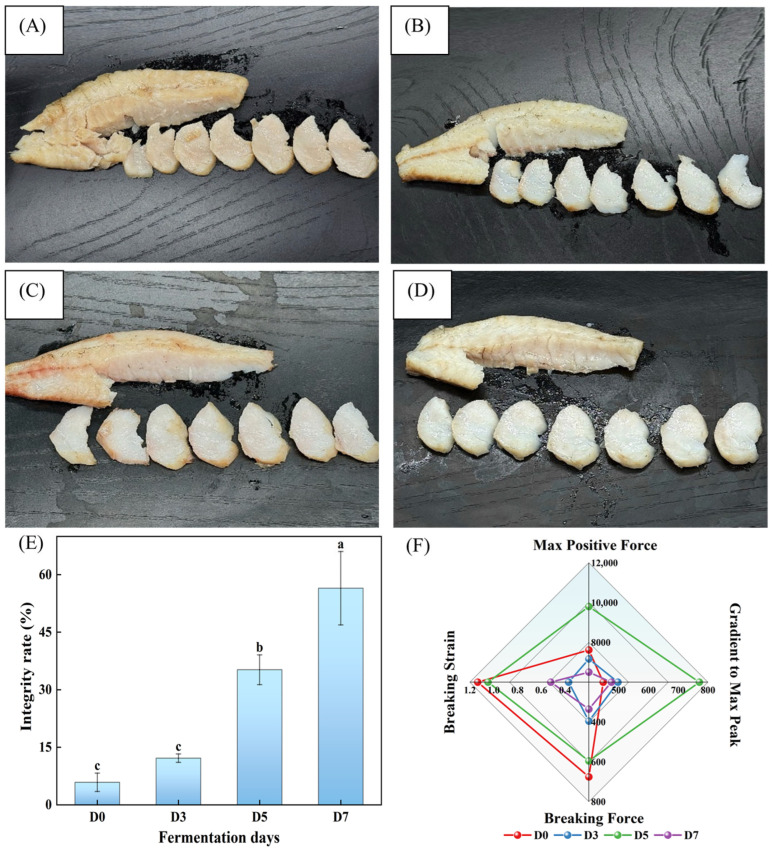
(**A**–**D**) correspond to the formation of GCMF in D0 (0-day fermentation), D3 (3 days fermentation), D5 (5 days fermentation), and D7 (7 days fermentation) samples, respectively; (**E**) shows the effect of salt concentration on the integrity rate of GCMF. (**F**) shows the effect of fermentation duration on the peeling of GCMF. Different letters on the bar chart indicate significant differences (*p* < 0.05).

To monitor the generation process of GCMF structure, the integrity of GCMF was evaluated during fermentation. As illustrated in Figure 4A, the structural integrity of GCMF in fermented fish meat progressively improved with prolonged fermentation time. For D0 samples, it is obvious that the separated fish segments appear fragmented with an incomplete GCMF structure, and the fish meat displays a lack of surface luster after the steaming process. After 3 days of fermentation, although the integrity rate of GCMF shows a slight increase to 12.18 ± 1.09%, the surface gloss and structural cohesion was significantly enhanced. Importantly, after 5 days of fermentation, the peeling of GCMF from fish products had only a subtle effect on the structure of the remaining fish meat. Moreover, the integrity rate of GCMF was determined to be 35.22 ± 3.88% and 56.48 ± 9.57%, respectively. Therefore, the results indicate that the fermentation process significantly facilitates the formation of garlic clove-like muscle flakes, and this effect is more pronounced with extended fermentation.

As shown in Figure S2, the fish meat flakes seemed to become increasingly separable and maintained better GCMF structural integrity as fermentation progressed. At the same time, during the 0–7 days of fermentation, the four mechanical parameters, including max positive force, gradient to max peak, breaking force, and breaking strain were presented in Figure 4F. At D0, the fish meat flakes were predominantly crushed and lacked the characteristic morphology of garlic cloves, which may be attributed to the high moisture content at this stage combined with the absence of external pressure, which led to dispersion of the myofibrillar structure. The values of the four mechanical parameters initially decreased at D3 but showed a rebound at D5. This phenomenon may be attributed to the activity of microorganisms or endogenous enzymes during fermentation, which degraded viscous substances. Upon heating, these degraded substances migrated to the surface, thereby enhancing the stability between muscle segments and increasing the resistance to separation. However, the parameters of max positive force, breaking force, and breaking strain obtained their lowest values at D7, suggesting the fish flakes were most peelable at this stage. This was likely because microbial activity or proteases further degraded collagen or myofibrillar proteins, weakening the intersegmental bonds and thus facilitating the separation of the fish flakes.

### 3.4. The Dynamic Changes in the Sensory, Textural, and Physiochemical Properties of the Garlic Clove-Structural Muscle Flakes (GCMF) During Fermentation

#### 3.4.1. Effect of Fermentation Duration on the Sensory Attribution of GCMF

As shown in Table 2, sensory scores for appearance showed a significant continuous increase with fermentation time of 7 days (*p* < 0.05), demonstrating enhanced evaluator satisfaction with the garlic-clove structure. This result is consistent with the above integrity and peeling properties evaluation. In addition, similar trends were observed for color and texture evaluation. The color scores increased from 11.38 ± 0.70 at D0 to 16.00 ± 1.00 at D7 (*p* < 0.05), while texture scores rose from 9.00 ± 1.41 to 15.88 ± 0.93 during the 7-day fermentation period (*p* < 0.05). These enhancements in sensory quality are likely be attributed to the hydrolysis by endogenous enzymes during fermentation, which may have facilitated the release and formation of sweet and umami peptides, as well as free amino acids, thereby improving the flavor profile of the fish [35]. Meanwhile, lipid and protein oxidation in the fermentation process may play a role in influencing sensory attributes.

#### 3.4.2. The Dynamic Changes in Textural Properties of GCMF During Fermentation

**Figure 5:**
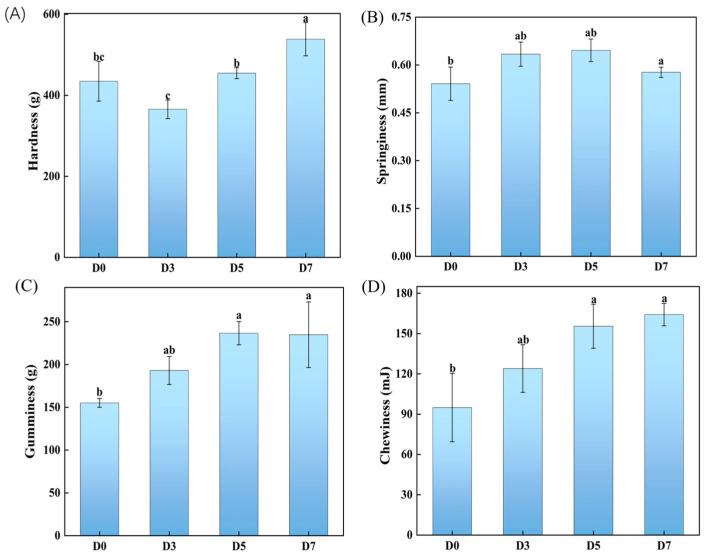
Effects of fermentation duration on TPA of GCMF, where (**A**–**D**) denote changes in fish meat hardness, springiness, gumminess, and chewiness, respectively. Different letters on the bar chart indicate significant differences (*p* < 0.05). D0: 0-day fermentation; D3: 3 days fermentation; D5: 5 days fermentation; D7: 7 days fermentation.

To evaluate the evolution of textural properties of GCMF during fermentation, the TPA experiment was carried out. As illustrated in Figure 5A, the hardness of the fish meat declined to its minimum value at D3, followed by a gradual increase from D3 to D7. In contrast, the springiness showed a trend of first increasing and then decreasing from D0 to D7 (Figure 5B). The reduction in hardness observed between D0 and D3 may be attributed to the dissolution or degradation of myofibrillar structures induced by NaCl and the degradation of protein by endogenous enzyme. Protein degradation significantly influences muscle texture and contributes to post mortem softening. The initial increase in springiness can be attributed to the decreased tenacity induced by collagen. The significant increase in fish hardness during the D3–D7 stage can be attributed to a dual mechanism. Firstly, the existence of salt and heat processing of samples promotes the formation of a more robust network structure in myofibrils. Secondly, mechanical pressure and protein denaturation and aggregation during the prolonged fermentation time led to a continuous decrease in water content and a considerable shrinkage and denser packing of the myofibrillar network, which leads to an increase in hardness and a decrease in springiness. Chewiness and gumminess showed a continuous rise throughout the fermentation period (Figure 5C,D). This phenomenon is likely due to the degradation of protein by endogenous enzyme or microbial fermentation. Then, these proteins subsequently formed a dense, continuous gel matrix upon heating, which coated the muscle fibers [36]. The synergistic effect of these processes collectively enhanced the chewiness and gumminess of GCMF.

#### 3.4.3. The Dynamic Changes in pH, Moisture Content, and TVB-N of GCMF During Fermentation

**Figure 6:**
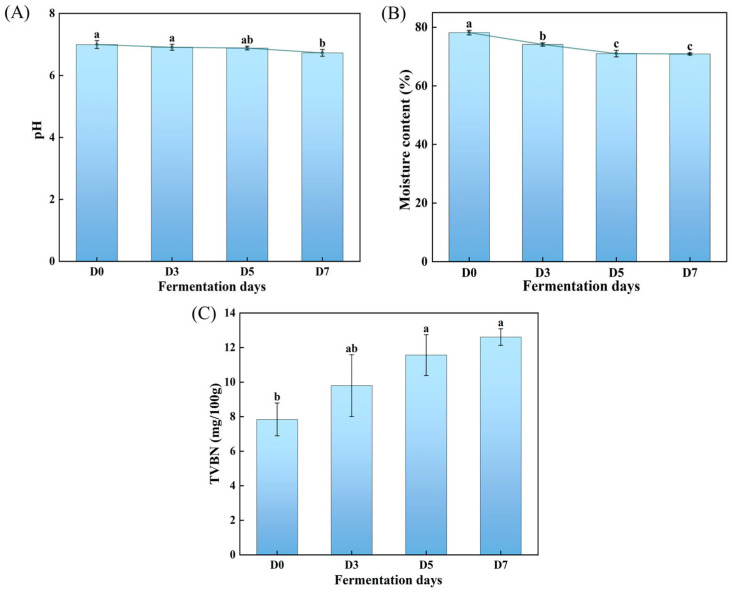
Effects of fermentation duration on the physicochemical properties of GCMF, where (**A**–**C**) correspond to changes in fish meat pH, moisture content, and TVBN, respectively. Different letters on the bar chart indicate significant differences (*p* < 0.05). D0: 0-day fermentation; D3: 3 days fermentation; D5: 5 days fermentation; D7: 7 days fermentation.

To explore the dynamic change in the physicochemical properties of GCMF during fermentation, the pH value, moisture content, and TVB-N were monitored. As shown in Figure 6A, the initial pH value of fresh fish meat was measured at 7.00 ± 0.13. After fermentation, the pH exhibits a significant decrease, with the lowest pH value of 6.73 ± 0.11 at D7. This phenomenon may be attributed to the accumulation of acidic metabolites produced during microbial decomposition. Generally, a lower pH value can significantly inhibit the growth of spoilage microorganisms [9], thereby facilitating quality control in the production of GCMF. Figure 6B illustrates the changes in moisture content of fish meat across different fermentation stages. A gradual reduction in moisture content was observed as fermentation progressed, reaching an equilibrium stage under 5 days of fermentation. The most significant moisture loss occurred within the first three days of fermentation, which can be attributed to pronounced dehydration caused by the initial salt-curing process under high salinity and mechanical pressure. After 5 days of fermentation, the moisture content reached a plateau, indicating that an internal concentration equilibrium had been achieved, beyond which only minimal free water remained available for further extraction. In addition, as a key indicator, TVB-N effectively reflects food freshness and safety by assessing the extent of protein decomposition and the formation of amino compounds [37]. As shown in Figure 6C, the TVB-N value in the fish meat increased with fermentation time, rising from 7.84 ± 0.95 mg/100 g on day 0 to 12.61 ± 0.48 mg/100 g by D7. This increase is attributed to the combined action of endogenous enzymes and microbial protein degradation [38,39]. According to current safety standard of China for animal-based aquatic products [40], the maximum allowable TVB-N value for salted aquatic products is 25 mg/100 g. Thus, the TVB-N value of fish fermented with 3% salt is significantly below the national standard, indicating the safety and quality of final products.

### 3.5. Dynamic Changes in Volatile Flavor Compounds During Fermentation

**Figure 7:**
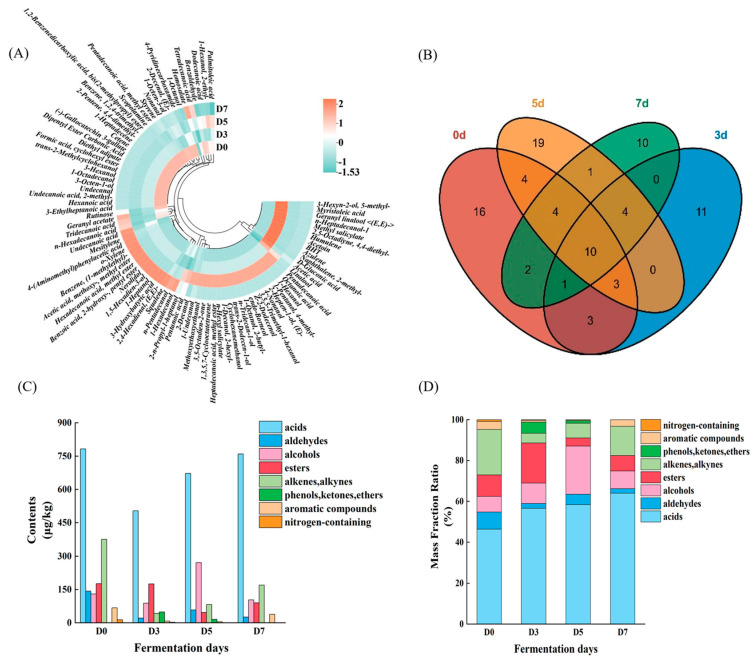
(**A**) circle heatmap of total volatile compounds in fish meat during fermentation; (**B**) Venn diagram of volatile flavor compounds in fish meat during fermentation; (**C**) volatile flavor compound content in fish meat during fermentation; (**D**) mass fraction ratio of volatile flavor compound types during fermentation. D0: 0-day fermentation; D3: 3 days fermentation; D5: 5 days fermentation; D7: 7 days fermentation.

To further explore the dynamic evolution of volatile flavor compounds during fermentation, a gas chromatography-mass spectrometer (GC-MS) experiment was carried out. As illustrated in Figure 7 and Table S2, a total of 88 volatile flavor compounds were identified throughout the fermentation process, categorized into 11 classes: 32 alcohols, 17 acids, 14 esters, 8 alkenes, 5 aromatic compounds, 5 aldehydes, 2 ketones, 2 nitrogen-containing compounds, 1 alkyne, 1 phenol, and 1 ether. These volatile flavor compounds may be generated by protein or lipid oxidation. Venn diagram analysis revealed that 10 of these compounds were consistently detected across all stages, implying that the fermentation process can significantly alter the volatile flavor compounds of mandarin fish (Figure 7B). More specifically, these volatile flavor compounds can be classified into eight primary categories: acids, aldehydes, alcohols, esters, alkenes/alkynes, phenols/ketones/ethers, aromatics, and nitrogen-containing compounds. Dynamic changes in volatile organic compounds profiles during fermentation were quantified through relative abundance and absolute content analyses (Figure 7C,D). The content of acids is always higher than that of other volatile flavor compounds, emerging as the predominant class of flavor compounds throughout the fermentation process of mandarin fish. Moreover, the relative proportion of acids showed an increasing trend throughout the fermentation process. This trend can be attributed to the concurrent decrease in aldehydes, alkenes/alkynes, and nitrogen-containing compounds. Notably, nitrogen-containing compounds declined steadily during fermentation and became undetectable by day 7. Generally, volatile nitrogen-containing compounds are usually associated with the development of undesirable flavors in fish, particularly fishy odors. Therefore, the reduction of nitrogen-containing compounds during the salt fermentation process is beneficial for the quality improvement of fermented products.

The concentration of volatile flavor compounds is considered to have a significant impact on the overall flavor. The contribution of volatile flavor compounds to the overall flavor of fermented fish can be evaluated based on their odor activity values (OAVs). As illustrated in Table 3, eight volatile compounds with OAV > 1 were identified during the fermentation of mandarin fish. These compounds include nonanal, undecanal, (E)-2-decenal, 1-hexanol, 1-octen-3-ol, linalool, methyl salicylate, and styrene. Notably, the OAVs of nonanal, 1-octen-3-ol, and styrene are significantly higher than that of other compounds, suggesting their predominant roles in shaping the flavor of fermented fish products.

Aldehydes are typical secondary products of lipid oxidation, particularly derived from the oxidative degradation of polyunsaturated fatty acids in muscle phospholipids, and play a crucial role in the development of fish smell [41,42]. These compounds are commonly associated with grassy, fatty, rancid, or floral aromas [43]. In this study, nonanal, undecanal, and (E)-2-decenal were identified as major aldehydes (OAV > 1), exhibiting fatty–citrus, oily–spicy, and floral–fruity flavor characteristics, respectively.

As shown in Figure 7C, the concentrations of aldehydes decreased during fermentation, indicating a gradual attenuation of lipid oxidation-related off-flavor compounds. Specifically, the content of nonanal declined markedly from 73.23 ± 9.94 μg/100 g at day 0 (D0) to 15.03 ± 5.70 μg/100 g at day 7 (D7) (Table S2), while undecanal was no longer detected after three days of fermentation. The reduction in aldehydes may be attributed to several factors, including the depletion of oxidation-susceptible lipids, further conversion of aldehydes into alcohols or acids, and the modulatory effects of microbial metabolism on lipid oxidation pathways during fermentation. 1-octen-3-ol, formed through the oxidation and degradation of highly unsaturated fatty acids, is commonly present in the volatile flavor profile of fish and contributes to characteristic fishy odors [44]. Its concentration decreased significantly from 30.92 ± 13.81 μg/100 g at D0 to 7.28 ± 4.32 μg/100 g at D7 (Table S2), further suggesting that fermentation effectively suppresses lipid oxidation-related undesirable volatiles. In contrast, linalool, characterized by floral and lavender-like aromas, may contribute positively to the overall flavor profile.

Overall, eight substances (nonanal, undecanal, (E)-2-decenal, 1-hexanol, 1-octen-3-ol, linalool, methyl salicylate, and styrene) with OAV > 1 were observed during fermentation. Notably, the contents of nonanal, 1-octen-3-ol, and undecanal were significantly decreased or eliminated, which demonstrated the positive effect on the decrease in undesirable flavors. These changes demonstrated the role of fermentation in reducing undesirable flavors compounds.

**Table 3:** Changes in OAV > 1 volatile flavor compounds during fermentation.

Volatile Compound	CAS.	Threshold ^a^ (μg/kg)	Odor Description ^b^	OAV > 1			
				D0	D3	D5	D7
Nonanal	124-19-6	1.10	fat, citrus, green	66.57 ± 26.42	8.14 ± 0.85	20.54 ± 4.59	13.66 ± 4.73
Undecanal	112-44-7	14.00	oil, pungent, sweet	1.23 ± 0.62	ND	ND	ND
2-Decenal, (E)-	3913-81-3	2.70	tallow	2.83 ± 1.33	0.49 ± 0.35	1.34 ± 0.11	0.98 ± 0.21
1-Hexanol	111-27-3	5.60	resin, flower, green	ND	ND	1.13 ± 0.86	ND
1-Octen-3-ol	3391-86-4	1.50	mushroom	21.01 ± 6.50	ND	8.69 ± 4.05	4.85 ± 2.84
Linalool	78-70-6	3.80	flower, lavender	ND	6.33 ± 3.59	5.61 ± 1.34	2.28 ± 0.97
Methyl salicylate	119-36-8	60.00	peppermint	0.00 ± 0.00	2.68 ± 0.85	ND	ND
Styrene	100-42-5	7.30	balsamic, gasoline	41.67 ± 17.32	3.61 ± 0.92	3.39 ± 3.95	9.48 ± 3.36

Note: ND indicates the substance was not detected; a. compound threshold reference [45]; b. odor descriptions primarily sourced from the online database: Flavornet (https://www.flavornet.org/flavornet.html, accessed on 18 December 2025).

## 4. Conclusions

This study demonstrated that salt-assisted curing fermentation can significantly improve fish meat quality while effectively reducing undesirable flavors. The results indicated that mandarin fish fermented with 3% NaCl concentration for 7 days exhibited the most distinct GCMF that was the easiest to separate, achieving a high integrity rate of 56.48 ± 9.57% and receiving the highest sensory evaluation scores. Analyses of the peeling properties and textural properties of GCMF revealed that the breaking force and maximum positive force required for GCMF separation decreased with increasing salt concentration and fermentation duration. In contrast, textural parameters such as hardness, chewiness, and gumminess increased correspondingly. A total of 88 volatile flavor compounds were identified during fermentation using HS-SPME-GC-MS analysis. Among these, eight compounds displayed OAV > 1: nonanal, undecanal, (E)-2-decenal, 1-hexanol, 1-octen-3-ol, linalool, methyl salicylate, and styrene. Notably, volatile compounds associated with a fishy odor decreased as fermentation progressed.

These findings provide a theoretical foundation for understanding the development of GCMF textural characteristics in fermented fish meat and offer insights into flavor regulation in fermented mandarin fish. Future studies should focus on elucidating the relationships between microbial succession and changes in volatile flavor compounds during fish fermentation, with the aim of identifying optimal starter cultures for fermented fish products. Such efforts would further improve and enhance the flavor quality and overall acceptability of fermented fish.

## Figures and Tables

**Table 1:** Effects of salt concentrations on the textural sensory evaluation of GCMF.

Sample	Organizational Form	Appearance	Taste
Y0	9.25 ± 1.71 ^c^	10.38 ± 2.39 ^c^	11.25 ± 2.49 ^c^
Y1	15.63 ± 3.60 ^ab^	15.38 ± 1.49 ^a^	14.13 ± 1.90 ^ab^
Y3	15.75 ± 1.85 ^a^	15.38 ± 1.87 ^a^	15.50 ± 1.80 ^a^
Y5	13.13 ± 2.32 ^b^	13.13 ± 2.42 ^b^	13.00 ± 2.00 ^bc^

Note: different letters in the same column indicate significant differences between groups (*p* < 0.05).

**Table 2:** Effect of fermentation duration on the textural sensory evaluation of GCMF.

Sample	Organizational Form	Appearance	Taste
D0	8.00 ± 1.22 ^c^	11.38 ± 0.70 ^c^	9.00 ± 1.41 ^d^
D3	8.13 ± 0.78 ^c^	12.00 ± 1.22 ^c^	11.38 ± 2.00 ^c^
D5	10.88 ± 1.62 ^b^	13.63 ± 1.41 ^b^	14.25 ± 1.71 ^b^
D7	15.88 ± 0.60 ^a^	16.00 ± 1.00 ^a^	15.88 ± 0.93 ^a^

Note: different letters in the same column indicate significant differences between groups (*p* < 0.05).

## Data Availability

The original contributions presented in this study are included in the article/Supplementary Materials. Further inquiries can be directed to the corresponding authors.

## Supplementary Materials

The following supporting information can be downloaded at: https://www.mdpi.com/article/10.3390/foods15030460/s1, Figure S1: (A–D) correspond to the changes in the peeling of GCMF for Y0 (without NaCl), Y1 (1% NaCl concentration), Y3 (3% NaCl concentration), and Y5 (5% NaCl concentration), respectively; Figure S2: (A–D) correspond to the changes in the peeling of GCMF for D0 (0-day fermentation), D3 (3 days fermentation), D5 (5 days fermentation), and D7 (7 days fermentation), respectively; Table S1: Structural and sensory evaluation criteria for GCMF; Table S2: Change in volatile compounds contents during fermentation.

## Abbreviations

The following abbreviations are used in this manuscript:

GCMF	Garlic clove-structural muscle flakes
Y0	0% NaCl concentration
Y1	1% NaCl concentration
Y3	3% NaCl concentration
Y5	5% NaCl concentration
D0	0-day fermentation
D3	3 days fermentation
D5	5 days fermentation
D7	7 days fermentation

## References

[1] Zhang G., Chu W., Hu S., Meng T., Pan L., Zhou R., Liu Z., Zhang J. (2011). Identification and Analysis of Muscle-Related Protein Isoforms Expressed in the White Muscle of the Mandarin Fish (*Siniperca chuatsi*). Mar. Biotechnol..

[2] Ahmed Z., Donkor O., Street W.A., Vasiljevic T. (2015). Calpains- and Cathepsins-Induced Myofibrillar Changes in Post-Mortem Fish: Impact on Structural Softening and Release of Bioactive Peptides. Trends Food Sci. Technol..

[3] Delbarre-Ladrat C., Chéret R., Taylor R., Verrez-Bagnis V. (2006). Trends in Postmortem Aging in Fish: Understanding of Proteolysis and Disorganization of the Myofibrillar Structure. Crit. Rev. Food Sci. Nutr..

[4] Li D.-Y., Huang Y., Wang K.-X., Dong X.-P., Yu D., Ge L.-H., Zhou D.-Y., Yu C.-X. (2018). Microstructural Characteristics of Turbot (Scophthalmus Maximus) Muscle: Effect of Salting and Processing. Int. J. Food Prop..

[5] Li C., Wu J., Li Y., Dai Z. (2013). Identification of the Aroma Compounds in Stinky Mandarin Fish (*Siniperca chuatsi*) and Comparison of Volatiles during Fermentation and Storage. Int. J. Food Sci. Technol..

[6] Gao P., Jiang Q., Xu Y., Xia W. (2018). Biosynthesis of Acetate Esters by Dominate Strains, Isolated from Chinese Traditional Fermented Fish (Suan yu). Food Chem..

[7] Thongsomboon W., Bunyatratchata A., Vongkampang T., Nammatra R., Prakitchaiwattana C., Siriamornpun S. (2023). Dynamic Changes in Thai-Style Fermented Fish: Low-Salt, Short Fermentation with Autochthonous Starter Culture. LWT-Food Sci. Technol..

[8] Tanabe K., Monguchi M., Inoue R., Zamami R., Nakanishi R., Manabe A., Oe K., Komatsuzaki N., Shima J. (2022). Lentilactobacillus Buchneri Domination during the Fermentation of Japanese Traditional Fermented Fish (Funazushi). Food Sci. Nutr..

[9] Gao P., Zhang X., Zhang Z., Jiang Q., Yang F., Yu P., Xia W., Liu S. (2024). Application of *Wickerhamomyces anomalus* and *Pichia fermentans* to Improve the Aroma of Fermented Sour Fish. LWT.

[10] Mao J., Wang X., Chen H., Zhao Z., Liu D., Zhang Y., Nie X. (2024). The Contribution of Microorganisms to the Quality and Flavor Formation of Chinese Traditional Fermented Meat and Fish Products. Foods.

[11] Shen Y., Wu Y., Wang Y., Li L., Li C., Zhao Y., Yang S. (2021). Contribution of Autochthonous Microbiota Succession to Flavor Formation during Chinese Fermented Mandarin Fish (*Siniperca chuatsi*). Food Chem..

[12] Yang Q., Xie J., Zhang Y., Tian Y., Song L., Zhao Y., Xiong J., Liu R., Rong J., Xiong S. (2025). Structural Evaluation and Formation Regularities of Garlic Clove-like Meat in Prefabricated Grass Carp. Food Chem..

[13] Zhou Y., Yang M., Yin J., Huang J., Yan Y., Zhang F., Xie N. (2023). Physicochemical Characteristics and Gel-Forming Properties of Mandarin Fish (*Siniperca chuatsi*) Protein during the Fish Fermentation with Lactobacillus Sake SMF-L5: The Formation of Garlic-Cloves Shaped Protein Gel. Food Chem..

[14] Yang Z., Liu S., Lv J., Sun Z., Xu W., Ji C., Liang H., Li S., Yu C., Lin X. (2020). Microbial Succession and the Changes of Flavor and Aroma in *Chouguiyu*, a Traditional Chinese Fermented Fish. Food Biosci..

[15] Guo K.-X., Hu B., Jiang Y., Li Z.-Y., Qi J., Yu M.-M. (2025). Comprehensive Insights into the Mechanism of Flavor Formation in Mandarin Fish (*Siniperca chuatsi*) with Inoculated Fermentation. Food Chem..

[16] Liu D., Liang L., Xia W., Regenstein J.M., Zhou P. (2013). Biochemical and Physical Changes of Grass Carp (*Ctenopharyngodon idella*) Fillets Stored at −3 and 0 °C. Food Chem..

[17] (2009). Sensory Analysis—General Guidance for the Design of Test Rooms.

[18] (2023). Sensory Analysis—General Guidelines for the Selection, Training and Monitoring of Selected Assessors and Expert Sensory Assessors.

[19] Szczesniak A.S., Kramer A., Szczesniak A.S. (1973). Instrumental Methods of Texture Measurements. Texture Measurements of Foods: Psychophysical Fundamentals: Sensory, Mechanical, and Chemical Procedures, and Their Interrelationships.

[20] Fan H., Luo Y., Yin X., Bao Y., Feng L. (2014). Biogenic Amine and Quality Changes in Lightly Salt- and Sugar-Salted Black Carp (*Mylopharyngodon piceus*) Fillets Stored at 4 °C. Food Chem..

[21] (2016). Nation Food Safety Standard Determination of Volatile Salt Nitrogen in Food.

[22] Gu J., Li S., Shen X., Liang Q., Xu T., Shi W. (2024). Effects of Different Fermenters on the Quality and Flavour of Fermented Mandarin Fish (*Siniperca chuatsi*). Int. J. Food Sci. Technol..

[23] Chen J.-N., Zhang Y.-Y., Huang X.-H., Dong M., Dong X.-P., Zhou D.-Y., Zhu B.-W., Qin L. (2023). Integrated Volatolomics and Metabolomics Analysis Reveals the Characteristic Flavor Formation in Chouguiyu, a Traditional Fermented Mandarin Fish of China. Food Chem..

[24] Hamm R. (1986). Functional properties of the myofibrillar system and their measurements. Muscle as Food.

[25] Parsons N., Knight P. (1990). Origin of Variable Extraction of Myosin from Myofibrils Treated with Salt and Pyrophosphate. J. Sci. Food Agric..

[26] Fang M.-C., Chin P.-S.-Y., Sung W.-C., Chen T.-Y. (2023). Physicochemical and Volatile Flavor Properties of Fish Skin under Conventional Frying, Air Frying and Vacuum Frying. Molecules.

[27] Li S., Lin S., Jiang P., Bao Z., Li S., Sun N. (2022). Insight into the Gel Properties of Antarctic Krill and Pacific White Shrimp Surimi Gels and the Feasibility of Polysaccharides as Texture Enhancers of Antarctic Krill Surimi Gels. Foods.

[28] Cai D., Li X., Liu H., Wen L., Qu D. (2024). Machine Learning and Flavoromics-Based Research Strategies for Determining the Characteristic Flavor of Food: A Review. Trends Food Sci. Technol..

[29] Yang W., Shi W., Qu Y., Wang Z., Shen S., Tu L., Huang H., Wu H. (2020). Research on the Quality Changes of Grass Carp during Brine Salting. Food Sci. Nutr..

[30] Guo J., Liu F., Gan C., Wang Y., Wang P., Li X., Hao J. (2022). Effects of Konjac Glucomannan with Different Viscosities on the Rheological and Microstructural Properties of Dough and the Performance of Steamed Bread. Food Chem..

[31] Sigurgisladottir S., Sigurdardottir M.S., Torrissen O., Vallet J.L., Hafsteinsson H. (2000). Effects of Different Salting and Smoking Processes on the Microstructure, the Texture and Yield of Atlantic Salmon (*Salmo salar*) Fillets. Food Res. Int..

[32] Bekhit A.E.-D.A., Holman B.W.B., Giteru S.G., Hopkins D.L. (2021). Total Volatile Basic Nitrogen (TVB-N) and Its Role in Meat Spoilage: A Review. Trends Food Sci. Technol..

[33] Liang Q., Hu X., Zhong B., Huang X., Wang H., Yu C., Tu Z., Li J. (2024). Regulating Effects of Low Salt Dry-Curing Pre-Treatment on Microbiota, Biochemical Changes and Flavour Precursors of Grass Carp (*Ctenopharyngodon idella*) Fillets during Storage at 4 °C. Food Chem. X.

[34] Szymczak M., Kołakowski E. (2016). Total Volatile Basic Nitrogen in Meat and Brine during Marinating of Herring. J. Aquat. Food Prod. Technol..

[35] Wu Y., Cao S.M. (2018). Study on Endogenous Protease and Protein Degradation of Dry-Salted *Decapterus maruadsi*. CyTA J. Food.

[36] Purslow P.P. (2018). Contribution of Collagen and Connective Tissue to Cooked Meat Toughness; Some Paradigms Reviewed. Meat Sci..

[37] Lannelongue M., Hanna M.O., Finne G., Nickelson R., Vanderzant C. (1982). Storage Characteristics of Finfish Fillets (*Archosargus probatocephalus*) Packaged in Modified Gas Atmospheres Containing Carbon Dioxide. J. Food Prot..

[38] Bian C., Yu H., Yang K., Mei J., Xie J. (2022). Effects of Single-, Dual-, and Multi-Frequency Ultrasound-Assisted Freezing on the Muscle Quality and Myofibrillar Protein Structure in Large Yellow Croaker (*Larimichthys crocea*). Food Chem. X.

[39] Sun Q., Sun F., Xia X., Xu H., Kong B. (2019). The Comparison of Ultrasound-Assisted Immersion Freezing, Air Freezing and Immersion Freezing on the Muscle Quality and Physicochemical Properties of Common Carp (*Cyprinus carpio*) during Freezing Storage. Ultrason. Sonochem..

[40] (2015). Nation Food Safety Standard for Animal based Aquatic Products.

[41] Huang L., Wu Z., Chen X., Weng P., Zhang X. (2018). Characterization of Flavour and Volatile Compounds of Fermented Squid Using Electronic Nose and HPMS in Combination with GC-MS. Int. J. Food Prop..

[42] Zeng X., Xia W., Jiang Q., Xu Y., Fan J. (2017). Contribution of Mixed Starter Cultures to Flavor Profile of Suanyu—A Traditional Chinese Low-Salt Fermented Whole Fish. J. Food Process. Preserv..

[43] Li Y., You S., Cheng L., Zeng H., Zheng B., Zhang Y. (2023). Physiochemical Quality, Microbial Diversity, and Volatile Components of Monascus-Fermented Hairtail Surimi. Foods.

[44] Li L., Xiao N., Li F., Pang Y., Yin Y., Sun Q., Shi W., Liu S. (2025). Unveiling the Dynamic Microbial Succession and Volatile Flavor Characteristic in Fermented Grass Carp during Fermentation with *Lactiplantibacillus plantarum*. J. Food Compos. Anal..

[45] van Gemert L.J. (2011). Compilations of Odour Threshold Values in Air, Water and Other Media.

