# Supplementary material for: Technological Optimization of Fermented Siniperca chuatsi Fish Processing Focused on Formation of Garlic Clove-Structural Muscle Flakes and Flavor Profiles
Source: Foods. 2026 Jan 28;15(3):460. doi: 10.3390/foods15030460 (PMC12896574; doi:10.3390/foods15030460)
Supplement: Supplementary file 1 [file foods-15-00460-s001.zip › foods-4081399-supplementary.pdf]

Table S1. Structural and sensory evaluation criteria for GCMF

Quality parameters	Description points	Score
GCMF tissue morphology	Severely damaged and uneven edges, obvious surface cracking of GCMF, irregular shape, no garlic clove-structural muscle flakes	1-6
	Moderately damaged and uneven edges, surface cracks present, the shape is generally well-formed, with a distinct garlic clove-structured muscle flakes.	7-10
	Mostly intact and smooth edges, few surface cracks, the shape is relatively uniform, with distinct garlic clove-structured muscle flakes.	11-14
	Intact and smooth edges, no surface cracks observed, regularly shaped, structurally sound	15-20
Appearance	Surface dry, color dull	1-6
	The surface appears relatively dry, and the color is somewhat dull.	7-10
	The surface is relatively moist and has a slight sheen.	11-14
	Surface is moist and glossy.	15-20
Texture	The meat is too loose or too tough, lacking springiness.	1-6
	The meat is relatively loose or firm, with a noticeable springiness.	7-10
	The meat is relatively loose or firm, with a springy texture.	11-14
	The meat is moderately firm and has a springy texture.	15-20

Table S2. Change in volatile compounds contents during fermentation.

Volatile Compound	CAS.	Content (μg/100g)			
		D0	D3	D5	D7
**Acids**					
Acetic acid	64-19-7	14.86±2.52	219.79±15.12	81.78±37.12	86.20±8.26
Pentanoic acid	109-52-4	2.06±0.67	ND	12.95±6.21	7.51±3.32
Hexanoic acid	142-62-1	2.20±0.41	ND	ND	ND
3-Ethylheptanoic acid	14272-47-0	2.09±0.30	ND	ND	ND
Octanoic acid	124-07-2	ND	ND	7.38±4.28	ND
Undecanoic acid, 2-methyl-	24323-25-9	5.22±1.14	ND	ND	ND
Dodecanoic acid	143-07-7	21.65±6.13	ND	33.28±9.86	ND
Undecanoic acid	112-37-8	ND	15.66±5.66	1.07±0.81	52.03±19.36
4-(Aminomethyl)phenylacetic acid	1200-05-1	ND	ND	2.28±1.39	42.37±8.37
Tridecanoic acid	638-53-9	11.54±1.70	8.65±2.48	11.11±4.51	30.62±9.74
Tetradecanoic acid	544-63-8	173.82±26.21	133.68±30.1	272.39±95.69	277.22±72.98
Myristoleic acid	544-64-9	ND	15.89±5.52	ND	ND
3-Hydroxybutyric acid	300-85-6	ND	ND	ND	31.36±10.87
Pentadecanoic acid	1002-84-2	ND	ND	119.89±55.98	ND
n-Hexadecanoic acid	57-10-3	69.82±16.78	62.76±13.43	ND	77.10±34.27
Palmitoleic acid	373-49-9	64.20±4.10	ND	ND	ND
D-Gluconic acid	526-95-4	0.86±0.15	ND	ND	ND
**aldehydes**					
Nonanal	124-19-6	73.23±9.94	8.96±0.94	22.59±5.11	15.03±5.70
2,4-Hexadienal, (E,E)-	142-83-6	ND	ND	ND	3.31±1.43
Benzaldehyde	100-52-7	23.49±7.57	10.58±8.60	30.93±8.58	ND
Undecanal	112-44-7	16.37±9.94	ND	ND	ND
2-Decenal, (E)-	3913-81-3	5.31±1.82	1.33±0.96	3.45±0.54	2.65±0.57
**alcohols**					
1-Hexanol	111-27-3	ND	ND	8.22±6.30	ND
1-Pentanol, 4-methyl-	626-89-1	ND	ND	9.15±5.24	ND
1-Octen-3-ol	3391-86-4	30.92±13.81	ND	14.46±6.95	7.28±4.32
1-Hexanol, 2-ethyl-	104-76-7	10.03±1.15	ND	22.47±19.10	ND
3-Octen-1-ol	18185-81-4	4.51±0.78	ND	ND	ND
1-Heptanol	111-70-6	ND	ND	ND	2.92±0.34
2-n-Propyl-1-heptanol	10042-59-8	18.28±8.76	ND	18.58±7.96	15.86±3.36
1,5-Hexadien-3-ol	924-41-4	ND	ND	ND	5.29±3.76
2-Decanol	1120-06-5	5.20±1.51	ND	15.40±3.12	ND
Linalool	78-70-6	ND	24.07±13.66	23.53±6.55	8.67±3.70
1-Octanol	111-87-5	6.17±0.86	2.46±0.59	ND	ND
2-Hepten-1-ol, (E)-	33467-76-4	ND	ND	4.12±1.48	ND
3-Hexyn-2-ol, 5-methyl-	23293-50-7	ND	ND	ND	ND
4-Nonanol	5932-79-6	ND	ND	10.87±4.91	ND

3,5,5-Trimethyl-1-hexanol	3452-97-9	ND	ND	6.92±7.53	ND
Z-2-Dodecenol	69064-36-4	ND	ND	6.11±8.42	ND
endo-Borneol	507-70-0	ND	ND	4.22±1.74	ND
1-Undecanol	112-42-5	2.58±0.56	2.27±0.61	21.10±13.56	6.07±2.05
1-Octanol, 2-butyl-	3913-02-8	ND	ND	ND	ND
Geranyl linalool <(E,E)->	1113-21-9	ND	ND	ND	ND
Nerolidol	142-50-7	ND	ND	ND	2.79±0.57
n-Tridecan-1-ol	112-70-9	ND	ND	6.84±4.74	ND
n-Pentadecanol	629-76-5	0.86±0.32	0.96±0.32	2.38±1.21	2.15±1.88
1-Hexadecanol	36653-82-4	1.60±0.78	ND	4.24±3.39	17.13±11.60
n-Heptadecanol-1	1454-85-9	ND	2.11±1.18	ND	ND
1-Octadecanol	112-92-5	0.27±0.10	ND	ND	ND
Rutinose	90-74-4	0.69±0.29	ND	ND	3.59±1.65
3-Hexanol	623-37-0	0.89±0.17	ND	ND	ND
trans-2-Dodecen-1-ol	69064-37-5	ND	ND	4.55±3.15	ND
Cyclohexanemethanol	100-49-2	ND	ND	23.16±8.92	ND
trans-2-Methylcyclohexanol	7443-52-9	1.81±0.60	ND	ND	ND
1-Decanol, 2-hexyl-	2425-77-6	ND	ND	11.97±10.41	ND
**esters**					
Formic acid, cyclohexyl ester	4351-54-6	28.83±4.91	ND	ND	ND
Methyl salicylate	119-36-8	ND	160.71±51.27	ND	ND
Geranyl acetate	105-87-3	2.80±0.37	ND	ND	5.64±1.80
Diethyl adipate	141-28-6	0.19±0.02	ND	ND	ND
Pentadecanoic acid, methyl ester	7132-64-1	14.11±4.06	10.08±1.39	10.61±3.51	11.91±4.98
Benzoic acid, 2-hydroxy-, pentyl ester	2050-08-0	ND	ND	ND	5.07±2.65
n-Hexyl salicylate	6259-76-3	ND	ND	6.01±3.54	ND
Heptadecanoic acid, methyl ester	1731-92-6	ND	ND	7.95±2.03	ND
Hexadecanoic acid, methyl ester	112-39-0	ND	ND	ND	9.39±4.10
Homosalate	118-56-9	ND	1.72±1.30	6.46±3.84	16.21±4.57
Dipentyl Ester Carbonic Acid	2050-94-4	0.31±0.23	ND	ND	ND
1,2-Benzenedicarboxylic acid, bis(2-methylpropyl) ester	84-69-5	20.48±3.38	9.49±2.09	ND	ND
Acetic acid, methoxy-, methyl ester	6290-49-9	ND	ND	ND	15.27±6.91
(-)-Gallocatechin 3-gallate	4233-96-9	2.95±0.62	ND	ND	ND
**alkenes**					
Styrene	100-42-5	396.53±59.00	26.33±6.71	28.71±7.18	69.21±26.13
Benzene, (1-methylethyl)-	98-82-8	ND	ND	ND	0.89±0.20
1,3,5,7-Cyclooctatetraene	629-20-9	ND	ND	15.66±10.90	ND
Humulene	6753-98-6	ND	3.65±4.92	ND	ND

Cetene	629-73-2	1.47±2.08	ND	ND	ND
1-Heptadecene	6765-39-5	5.37±3.97	ND	ND	ND
2-Pentene, 4,4-dimethyl-	26232-98-4	1.70±1.21	ND	ND	ND
Squalene	111-02-4	5.91±1.32	9.77±2.55	37.94±6.12	26.54±12.90
**aromatic compounds**					
o-Xylene	95-47-6	ND	ND	ND	2.59±0.93
Mesitylene	108-67-8	ND	2.42±1.62	4.31±1.35	61.48±18.48
Benzene, 1,2,4-trimethyl-	95-63-6	55.30±17.96	ND	ND	ND
Azulene	275-51-4	ND	1.49±0.46	ND	ND
Naphthalene, 2-methyl-	91-57-6	ND	3.91±2.18	ND	ND
**ketones**					
Acetoin	513-86-0	ND	1.54±0.30	ND	ND
3,5-Octadien-2-one	38284-27-4	ND	ND	13.89±1.70	ND
**nitrogen-containing compounds**					
Scopolamine	138-12-5	2.82±0.50	ND	0.08±0.05	ND
4-Pyridinecarboxamide	1453-82-3	2.39±0.19	2.14±0.81	ND	ND
**alkyne**					
2,5-Octadiyne, 4,4-diethyl-	61227-87-0	ND	2.42±1.09	ND	ND
**phenol**					
BHT	128-37-0	ND	47.01±65.97	ND	ND
**ether**					
Methoxyethoxyethanol	111-77-3	ND	ND	1.27±1.01	ND
Total		1095.29±52.47	893.67±18.75	1335.36±391.61	1359.48±292.96

Note: ND indicates the substance was not detected

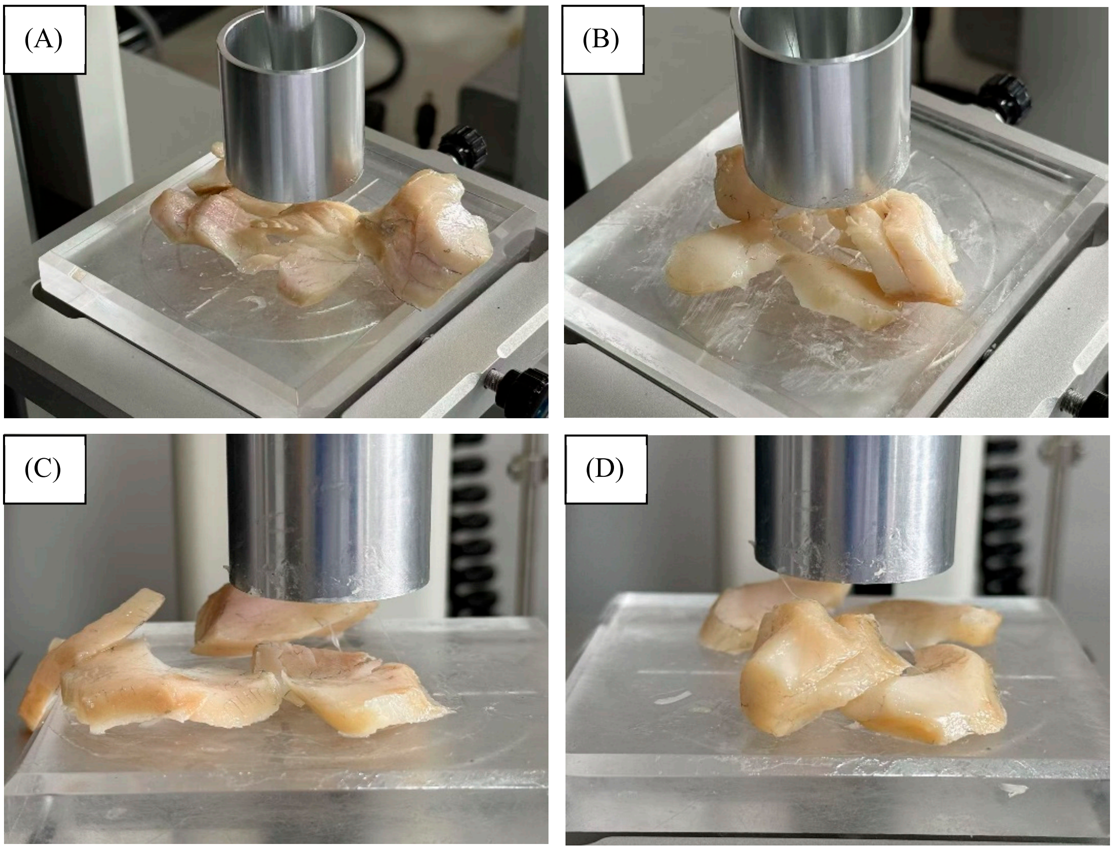

Figure S1. (A–D) correspond to the changes in the peeling of GCMF for Y0 (without NaCl), Y1 (1% NaCl concentration), Y3 (3% NaCl concentration), and Y5 (5% NaCl concentration), respectively.

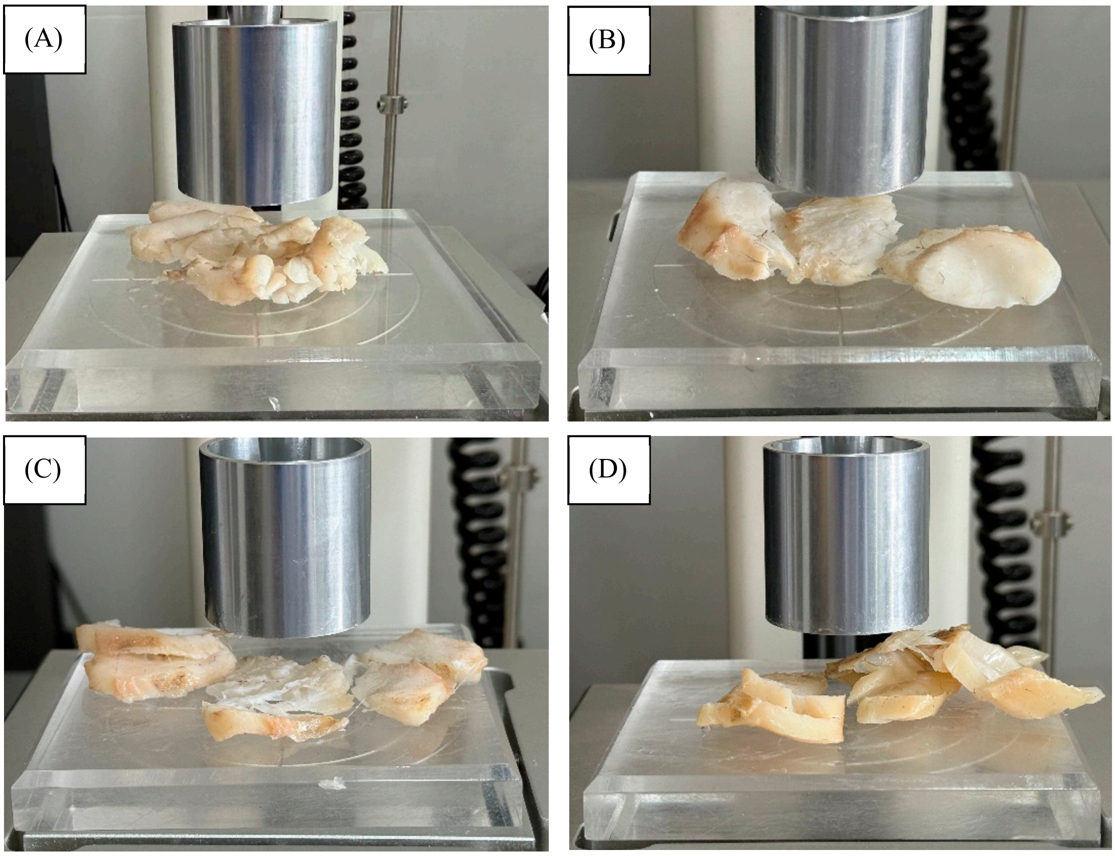

Figure S2. (A–D) correspond to the changes in the peeling of GCMF for D0 (0 day fermentation), D3 (3 days fermentation), D5 (5 days fermentation), and D7 (7 days fermentation), respectively.
